# First trimester exposure to ambient gaseous air pollutants and risk of orofacial clefts: a case–control study in Changsha, China

**DOI:** 10.1186/s12903-021-01876-7

**Published:** 2021-10-15

**Authors:** Wen Jiang, Wanqin Xie, Bin Ni, Haiyan Zhou, Zhiyu Liu, Xingli Li

**Affiliations:** 1 Department of Epidemiology and Health Statistics, Xiangya School of Public Health, Central South University, Xiangya Road, Kaifu District, Changsha, 410078 China; 2 Maternal and Child Health Care Hospital of Hunan Province, Changsha, China

**Keywords:** Gaseous air pollution, Particulate matter, Orofacial clefts

## Abstract

**Background:**

A growing body of studies have investigated the association between air pollution exposure during early pregnancy and the risk of orofacial clefts, but these studies put more emphasis on particulate matter and reported inconsistent results, while research on the independent effects of gaseous air pollutants on orofacial clefts has been quite inadequate, especially in China.

**Methods:**

A case–control study was conducted in Changsha, China from 2015 to 2018. A total of 446 cases and 4460 controls were included in the study. Daily concentrations of CO, NO_2_, SO_2_, O_3_, PM_2.5_ and PM_10_ during the first trimester of pregnancy were assigned to each subject using the nearest monitoring station method. Multivariate logistic regression models were applied to evaluate the associations of monthly average exposure to gaseous air pollutants with orofacial clefts and its subtypes before and after adjusting for particulate matter. Variance inflation factors (VIFs) were used to determine if the effects of gaseous air pollutants could be independent of particulate matter.

**Results:**

Increase in CO, NO_2_ and SO_2_ significantly increased the risk of cleft lip with or without cleft palate (CL/P) in all months during the first trimester of pregnancy, with aORs ranging from 1.39 to 1.48, from 1.35 to 1.61 and from 1.22 to 1.35, respectively. The risk of cleft palate only (CPO) increased with increasing NO_2_ exposure levels in the first trimester of pregnancy, with aORs ranging from 1.60 to 1.66. These effects sustained and even exacerbated after adjusting for particulate matter. No significant effect of O_3_ was observed.

**Conclusions:**

Our study suggested that maternal exposure to CO, NO_2_, and SO_2_ during the first trimester of pregnancy might contribute to the development of orofacial clefts, and the associations were potentially independent of particulate matter.

**Supplementary Information:**

The online version contains supplementary material available at 10.1186/s12903-021-01876-7.

## Introduction

Orofacial clefts are common congenital malformations comprising a range of disorders affecting the lips and oral cavity. They are generally subdivided into two types according to distinct developmental origins from embryo: cleft palate only (CPO) and cleft lip with or without cleft palate (CL/P) [1]. The malformations occur in about 1.7 per 1000 live-born babies, which can increase morbidity and mortality of perinatal infants, impair social adaptive ability of the survivors, and impose financial burdens on the family involved and the society at large because of the relevant health care services required [2]. Both genetic components and environmental factors have been linked to the development of orofacial clefts. However, the etiology of the defects is not fully understood [3].

Animal studies have shown that pregnant mice suffering from higher levels of carbon monoxide (CO) and ozone (O_3_) tended to give birth to more offspring with skeletal malformations [4, 5], which may be the consequence of oxidative stress, cell toxicity, and hemodynamics during organogenesis period [6]. These evidence and potential biological rationales have encouraged a growing body of epidemiological studies to assess possible associations of air pollutants exposure during early pregnancy with congenital anomalies, which include orofacial clefts [7–10]. However, most previous studies were conducted in developed countries, where the concentration levels of air pollution tend to be lower than those in developing countries. Besides, existing researches put more emphasis on particulate matter (PM), while attention given to toxic gaseous components of air pollutants, such as CO, nitrogen dioxide (NO_2_), sulfur dioxide (SO_2_) and O_3_ has been limited, with positive associations found in some [6, 11] but null in others [12, 13]. More importantly, air pollution consists of various solid particles and gases, and it is unclear whether the associations between gaseous air pollutants and orofacial clefts are independent or are or due to exposure to other air pollutants.

Over the past decades, China has achieved great economical development. Meanwhile, the largest developing country in the world has been confronted with increasing challenges in environmental protection [14], and the health effects of gaseous air pollutants, which originate from massive combustion of various fuels, are one of the greatest concerns. Based on our review of existing literature, however, only a few studies have explored the effects of exposure to gaseous air pollutants on orofacial clefts in China, but have reported inconsistent results [15–18]. Of these studies, two used single-pollutant model without adjustment for the coexisting pollutants, thus failed to isolate the independent role of gaseous air pollutants [15, 16]. Besides, it has been proven that the concentration of air pollution varies greatly in different areas of China [19], which may lead to different health effects. Therefore, more studies on the associations between gaseous air pollutants exposure and orofacial clefts are needed, especially in areas of high incidence of birth defects without exact causes, such as Changsha, China [20].

In this study, we utilized maternal and child health monitoring data from Changsha, China during the 2015–2018 period to examine the independent effects of maternal gaseous air pollutants (CO, NO_2_, SO_2_ and O_3_) exposure during early pregnancy on the risk of orofacial clefts.

## Methods

### Study area

Changsha is a subtropical city with a typical monsoon climate located in the middle and lower reaches of the Yangtze River, central China, where the climate characterized by hot and rainy in summer, mild and light rain in winter. Local season can be divided into warm (May–October) and cold (November to April) based on temperature variation. The annual average precipitation is approximately 1361.6 mm, and the annual average temperature is 17.2 °C. It is also the capital city and the economic and cultural central of Hunan Province. According to statistics, approximately 8.39 million people reside in a land area of 11,819.0 km^2^ in Changsha.

### Study population

This is a case–control study. The cases and controls were enrolled from the hospital based birth defect monitoring (HBBDM) system of Hunan Province and the electronic medical records (EMR) system of Maternal and Child Health Care Hospital of Hunan Province, respectively. Details of the establishment and operation of the two systems were described elsewhere [20]. Briefly, we abstracted all records of orofacial clefts (including stillbirth, dead fetus and live birth) with maternal residence during early pregnancy in Changsha and estimated date of conception between 1 January 2015 and 31 December 2018 from HBBDM system (n = 589). For each mother-infant pair, we collected information regarding maternal age, residential address during early pregnancy, education level, gravidity, date of pregnancy termination, gestational weeks (weeks + days), health condition, infant sex and subtype classification of orofacial clefts. Cases were excluded if they met any of the following criteria: (1) mother-infant pair information mentioned above missing from the system (n = 20); (2) maternal illness conditions, including gestation weeks < 20 or > 44, gestational diabetes, a family history of orofacial clefts or a history of other illness during pregnancy [i.e. fever (> 38 °C), infection or exposure to antibiotics] (n = 18); (3) with simultaneous genetic anomalies other than orofacial clefts (n = 12). Specifically, given that the regional nature of the studied air pollutants concentrations are different from each other, which may contribute to unacceptable exposure misclassification for those air pollutants with larger spatial gradients when using individual exposure assessment method listed below, a maximum radius of 25 km from the nearest monitoring station was selected as threshold to include cases following a previous study [21] (93 cases excluded). At the same time, 60,000 records of live-born infants without any congenital anomalies within the same range of estimated date of conception and maternal residences were randomly selected from the EMR system, followed by an exclusion process using the same criteria for cases. After that, remaining mother-infant pairs from the EMR system were matched 1:10 to cases by year of conception as controls. Date of conception was estimated using the termination date of pregnancy minus gestational age (week + day) for each mother.

### Classification of orofacial clefts

Cases of orofacial clefts were diagnosed according to the International Classification of Diseases-10 (ICD-10) codes, which including cleft palate only (Q35), cleft lip without cleft palate (Q36) and cleft lip with cleft palate (Q37), and the later two categories were merged into one for further analysis.

### Exposure assessment

It has been well documented that the development of the lip and palate entails a complex series of events that happen between the 4th and 12th week of gestation [1, 6], we thus chose the first trimester of pregnancy as the susceptibility window for the collection of air pollutants data.

**Fig. 1:**
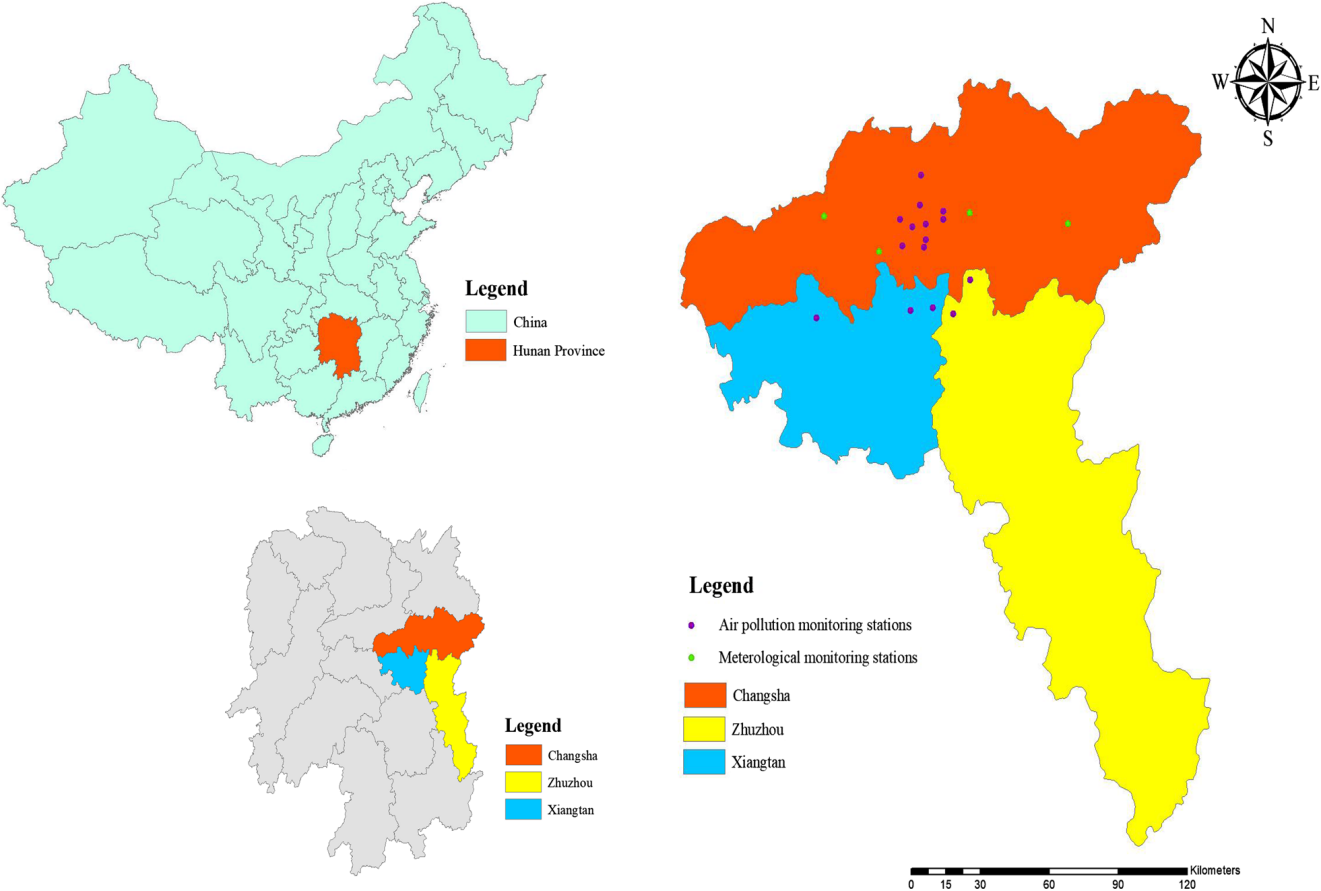
Location of monitoring stations included in the study. There are 15 air pollution monitoring stations and 4 meteorological monitoring stations. The 15 air pollution monitoring stations consist of 10 from Changsha, 3 from Xiangtan, and 2 from Zhuzhou, respectively

The pollution data were obtained from Hunan Environmental Monitoring Center. We included 15 air pollution monitoring stations in the study, with 10 from Changsha, 3 from Xiangtan, and 2 from Zhuzhou, respectively. The last 5 monitoring stations were included because they are in close vicinity of Changsha, and thus could be used to estimate the exposure levels of cases and controls who lived nearby (Fig. 1). All of these 15 monitoring stations are national air-quality monitoring stations. The installation of these stations and their collection of air pollutants strictly follow the national regulations. Daily 24-h mean concentrations of CO, NO_2_, SO_2_, PM_2.5_, and PM_10_, and 8-h maximum concentration of ozone during the period of 2015–2018 were collected to satisfy the requirements of our analysis.

Individual daily exposure levels to air pollutants during the first trimester of pregnancy were predicted using the nearest monitoring station approach in regard of maternal residence. Briefly, we geocoded the residential address of each participating mother during early pregnancy and the locations of monitoring stations into longitude and latitude by using Baidu Maps (https://map.baidu.com), and calculated the distance between monitoring stations and each residential address using the software ARCGIS (version 10.3). Air pollutants data from the nearest monitoring station during the susceptibility window was then linked to each participating mother as the estimates of individual exposure levels. Monthly average values of CO, NO_2_, SO_2_, O_3_, PM_2.5_, and PM_10_ were calculated for each subject.

To control for potential confounding by weather conditions, data on daily mean temperature and mean relative humidity of Changsha during the same study period from 4 local meteorological factors monitoring stations were averaged and used to represent the individual exposure levels during early pregnancy.

### Statistic analysis

We assessed the association between monthly average exposure levels of gaseous air pollutants with orofacial clefts using multivariate logistic regression models. The whole analysis procedure in the study was divided into two stages. In the first stage, we constructed single-pollutant models for overall cases and subtypes. In the models of overall cases and CL/P, we adjusted for the following confounding factors according to the existing literatures and the significant differences in population characteristics between cases and controls: maternal age (classified as < 20 years old, 20–24 years old, 25–29 years old and ≥ 30 years old), maternal educational level (classified as middle school or below, high/technical school, and college or higher degree), gravidity (classified as 1, 2, 3 and ≥ 4), infant sex (classified as female and male), plurality (classified as singleton and multiple birth), temperature and relative humidity. In the CPO model, we adjusted for same confounding factors except for sex because no significant sexual difference between CPO cases and controls was found. In the second stage, we further adjusted for PM_2.5_ and PM_10_ in separate two-pollutant models. In order to examine if PM could be adjusted in the models, variance inflation factors (VIFs) were calculated, where a VIF > 10 would suggest the existence of collinearity in the two-pollutant models, meaning that the confounding effect of PM_2.5_/PM_10_ could not be simultaneously adjusted. In contrast; a VIF ≤ 10 would indicate no significant collinearity, so the effects of gaseous air pollutants could be considered as independent. ORs, along with their 95% confidence intervals (CIs), were used to present the effects of per interquartile range (IQR) increase in CO, NO_2_, SO_2_ and O_3_, separately. Moreover, considering that cases with multiple congenital anomalies might have more complex causes and a different etiology, which possibly bias our findings [22, 23], the two analysis stages which only included isolated orofacial cleft cases were conducted as a sensitivity analysis to test the stability of the results in the base case analysis.

All analyses were performed with the use of SAS (SAS Institute, Inc., Cary, North Carolina) and R (version 4.0.0; R Development Core Team), and a two tailed *P* value less than 0.05 was considered statistically significant.

## Results

### Descriptive statistics

A total of 446 cases of orofacial clefts and 4460 controls were included in the current analysis. Compared to controls, cases had a higher ratio of mothers with younger age, lower educational levels and a history of multiple gravidity. Besides, difference in gender structure between CL/P cases and controls was obvious, with higher ratio of male in cases. No significant difference in the season of conception was observed. Detailed characteristics of the subjects are summarized in Table 1. Additionally, the median (25 percentile to 75 percentile) distance between maternal residences during early pregnancy and the nearest monitoring station was 4.14 (2.56–10.61) km.

**Table 1:** Characteristics of cases and controls from Changsha included in the study, 2015–2018

Characteristics	Controls (n = 4460)		Overall Cases (n = 446)		*P* value^a^	Cleft palate only (n = 75)	
	N	(%)	N	(%)		N	(%)
Maternal age (years)					< 0.0001		
< 20	14	0.31	5	1.12		0	0.00
20–24	363	8.14	68	15.25		12	16.00
25–29	1982	44.44	189	42.38		33	44.00
≥ 30	2101	47.11	184	41.26		30	40.00
Maternal education					< 0.0001		
Middle school or below	0	0.00	60	13.45		10	13.33
High/technical school	1392	31.21	128	28.70		18	24.00
College or higher degree	3068	68.79	258	57.85		47	62.67
Gravidity					< 0.0001		
1	1559	34.96	106	23.77		21	28.00
2	1315	29.48	131	29.37		24	32.00
3	799	17.91	101	22.65		12	16.00
≥ 4	787	17.65	108	24.22		18	24.00
Season of conception					0.0742		
Warm	2615	58.63	242	54.26		44	58.67
Cold	1845	41.37	204	45.74		31	41.33
Plurality					0.1397		
Singleton	4252	93.95	432	96.86		74	98.67
Multiple birth	208	6.05	14	3.14		1	1.33
Infant sex^b^					0.0004		
Female	2135	47.62	174	39.01		36	48.00
Male	2325	52.38	272	60.99		39	52.00

^a^Chi-square test for equal distribution of categorical variables between overall cases and controls

^b^There is no significant sexual difference between CPO cases and controls

The distribution of daily average air pollutant concentrations and meteorological factors in Changsha varied greatly during the study period, with a median (25th to 75th percentile range) concentration of 0.85 (0.71–1.04) mg/m^3^ for CO, 31.30 (23.20–44.70) μg/m^3^ for NO_2_, 12.60 (9.00–17.00) μg/m^3^ for SO_2_, 84.55 (54.55–117.50) μg/m^3^ for O_3_, 44.20 (28.50–67.00) μg/m^3^ for PM_2.5_, 66.50 (46.22–96.40) μg/m^3^ for PM_10_, 18.68 (10.45–25.18) °C for temperature and 82.00 (72.00–91.50)% for relative humidity, respectively (Table 2). The results of correlation analysis of the air pollutants are presented in Additional file 1: Table S1, showing that all air pollutants were significantly associated with one another (*P* < 0.001), with Spearman correlation coefficients ranging from − 0.08 to 0.91.

**Table 2:** Distribution of daily average air pollutant concentration and meteorological factors during 2015–2018 in Changsha, China

	Min	25 percentile	Median	75 percentile	Max	IQR	Mean
CO (mg/m^3^)	0.41	0.71	0.85	1.04	2.17	0.33	0.90
SO_2_ (μg/m^3^)	3.80	9.00	12.60	17.00	71.20	8.00	14.04
NO_2_ (μg/m^3^)	9.90	23.20	31.30	44.70	107.00	21.50	35.56
O_3_ (μg/m^3^)	7.70	54.55	84.55	117.50	228.20	62.95	88.51
PM_2.5_ (μg/m^3^)	3.20	28.50	44.20	67.00	278.90	38.50	52.26
PM_10_ (μg/m^3^)	5.40	46.22	66.50	96.40	411.30	50.18	75.40
Temperature (°C)	− 1.98	10.45	18.68	25.18	33.03	14.73	18.04
Relative humidity (%)	40.25	72.00	82.00	91.50	99.25	19.50	80.61

### Associations between gaseous air pollutants and orofacial clefts

Table 3 shows the aORs and corresponding 95% confidence intervals (CIs) for the risk of orofacial clefts in relation to CO, NO_2_, SO_2_ and O_3_ exposure during the first trimester of pregnancy. Elevated risks of overall cases for an IQR increase in CO, NO_2_ and SO_2_ were found in all months of the first trimester of pregnancy, with aORs ranging from 1.35 to 1.42 for CO, from 1.37 to 1.58 for NO_2_, and from 1.21 to 1.31 for SO_2_, respectively. By contrast, monthly O_3_ exposure showed nonsignificant effects on overall cases. In the subgroup analysis, similar association patterns for the outcome CL/P were observed. However, the risk of CPO only increased with increasing NO_2_ exposure levels in the first trimester of pregnancy, with aORs ranging from 1.60 to 1.66.

**Table 3:** Adjusted odds ratios (95% confidence intervals) for orofacial clefts during the first trimester of pregnancy

Air pollutant/time scale	Overall cases (n = 446)	Cleft lip with or without cleft palate (n = 371)	Cleft palate only (n = 75)
	aOR (95% CI)	aOR (95% CI)	aOR (95% CI)
Per IQR increase in CO			
1st month	1.37 (1.19, 1.59)	1.44 (1.23, 1.69)	1.24 (0.84, 1.82)
2nd month	1.35 (1.17, 1.57)	1.39 (1.19, 1.63)	1.19 (0.81, 1.76)
3rd month	1.42 (1.23, 1.64)	1.48 (1.26, 1.73)	1.16 (0.79, 1.68)
Per IQR increase in NO_2_			
1st month	1.58 (1.36, 1.83)	1.606 (1.36, 1.89)	1.63 (1.12, 2.37)
2nd month	1.52 (1.31, 1.77)	1.48 (1.25, 1.75)	1.66 (1.15, 2.39)
3rd month	1.37 (1.18, 1.59)	1.35 (1.14, 1.59)	1.60 (1.12, 2.29)
Per IQR increase in SO_2_			
1st month	1.21 (1.03, 1.41)	1.22 (1.04, 1.44)	1.20 (0.77, 1.85)
2nd month	1.31 (1.12, 1.55)	1.35 (1.14, 1.61)	1.16 (0.73, 1.84)
3rd month	1.30 (1.12, 1.51)	1.34 (1.14, 1.58)	0.99 (0.66, 1.47)
Per IQR increase in O_3_			
1st month	0.85 (0.70, 1.03)	0.84 (0.68, 1.03)	0.91 (0.56, 1.46)
2nd month	0.91 (0.95, 1.10)	0.90 (0.73, 1.11)	0.86 (0.54, 1.37)
3rd month	1.04 (0.86, 1.26)	1.08 (0.87, 1.34)	0.89 (0.56, 1.41)

In the models of overall cases and CL/P, adjusted covariates including maternal age, maternal educational level, gravidity, infant sex, plurality, temperature and relative humidity. In the model of CPO, only maternal age, maternal educational level, gravidity, plurality, temperature and relative humidity were adjusted

aOR, adjusted odds ratio; CI, confidence interval

Table 4 presents the associations between outcomes and four gaseous air pollutants after adjusting for PM_2.5_. Most of the significant effects were sustained and even enhanced, with aORs ranging from 1.46 to 1.91 for CO, 1.60 to 2.11 for NO_2_, 1.34 to 2.10 for SO_2_, respectively. Enhanced effects on orofacial clefts were also observed for CO, NO_2_ and SO_2_ after adjusting for PM_10_ (Table 5). All VIFs in the two-pollutant models were less than 2 (data not shown), suggesting there is no evidence for significant collinearity.

**Table 4:** Adjusted odds ratios (95% confidence intervals) for orofacial clefts after additionally adjusting for PM_2.5_ during the first trimester of pregnancy

Air pollutant/time scale	Overall cases (n = 446)	Cleft lip with or without cleft palate (n = 371)	Cleft palate only (n = 75)
	aOR (95% CI)	aOR (95% CI)	aOR (95% CI)
Per IQR increase in CO			
1st month	1.46 (1.20, 1.77)	1.61 (1.30, 2.00)	0.95 (0.56, 1.61)
2nd month	1.48 (1.22, 1.80)	1.57 (1.28, 1.94)	1.06 (0.62, 1.80)
3rd month	1.79 (1.48, 2.17)	1.91 (1.55, 2.36)	1.14 (0.66, 1.96)
Per IQR increase in NO_2_			
1st month	1.92 (156, 2.36)	2.00 (1.59, 2.51)	1.88 (0.94, 2.69)
2nd month	1.84 (1.50, 2.26)	1.76 (1.41, 2.21)	2.05 (1.22, 3.44)
3rd month	1.65 (1.36, 2.01)	1.60 (1.29, 1.99)	2.11 (1.28, 3.47)
Per IQR increase in SO_2_			
1st month	1.46 (0.91, 2.33)	1.18 (0.98, 1.42)	1.00 (0.60, 1.64)
2nd month	2.00 (1.25, 3.19)	1.34 (1.12, 1.62)	1.07 (0.65, 1.76)
3rd month	2.10 (1.37, 3.21)	1.36 (1.15, 1.62)	0.95 (0.63, 1.45)
Per IQR increase in O_3_			
1st month	0.94 (0.74, 1.18)	0.91 (0.70, 1.17)	1.23 (0.70, 2.17)
2nd month	0.99 (0.78, 1.26)	0.97 (0.75, 1.26)	1.00 (0.57, 1.78)
3rd month	1.12 (0.88, 1.42)	1.18 (0.91, 1.54)	0.94 (0.53, 1.68)

In the models of overall cases and CL/P, adjusted covariates including maternal age, maternal educational level, gravidity, infant sex, plurality, temperature, relative humidity and PM_2.5_. In the model of CPO, only maternal age, maternal educational level, gravidity, plurality, temperature, relative humidity and PM_2.5_ were adjusted

aOR, adjusted odds ratio; CI, confidence interval

**Table 5:** Adjusted odds ratios (95% confidence intervals) for orofacial clefts after additionally adjusting for PM_10_ during the first trimester of pregnancy

Air pollutant/time scale	Overall cases (n = 446)	Cleft lip with or without cleft palate (n = 371)	Cleft palate only (n = 75)
	aOR (95% CI)	aOR (95% CI)	aOR (95% CI)
Per IQR increase in CO			
1st month	1.41 (1.20, 1.67)	1.49 (1.25, 1.78)	1.23 (0.80, 1.91)
2nd month	1.36 (1.16, 1.60)	1.40 (1.18, 1.67)	1.16 (0.74, 1.81)
3rd month	1.49 (1.27, 1.75)	1.55 (1.30, 1.84)	1.17 (0.76, 1.81)
Per IQR increase in NO_2_			
1st month	1.78 (1.49, 2.12)	1.79 (1.48, 2.18)	1.88 (1.19, 2.96)
2nd month	1.66 (1.39, 2.00)	1.59 (1.30, 1.94)	1.98 (1.25, 3.14)
3rd month	1.50 (1.26, 1.79)	1.44 (1.18, 1.75)	1.98 (1.28, 3.07)
Per IQR increase in SO_2_			
1st month	1.20 (1.01, 1.44)	1.21 (1.00, 1.46)	1.17 (0.71, 1.94)
2nd month	1.31 (1.09, 1.58)	1.35 (1.11, 1.65)	1.09 (0.63, 1.88)
3rd month	1.35 (1.14, 1.60)	1.39 (1.16, 1.67)	0.96 (0.60, 1.54)
Per IQR increase in O_3_			
1st month	0.87 (0.71, 1.06)	0.86 (0.69, 1.06)	0.93 (0.57, 1.52)
2nd month	0.94 (0.78, 1.14)	0.94 (0.76, 1.16)	0.89 (0.55, 1.43)
3rd month	1.06 (0.87, 1.30)	1.11 (0.89, 1.39)	0.89 (0.55, 1.43)

In the models of overall cases and CL/P, adjusted covariates including maternal age, maternal educational level, gravidity, infant sex, plurality, temperature, relative humidity and PM_10_. In the model of CPO, only maternal age, maternal educational level, gravidity, plurality, temperature, relative humidity and PM_10_ were adjusted

aOR, adjusted odds ratio; CI, confidence interval

In the sensitivity analysis, the associations between gaseous air pollutants exposure and the risk of orofacial clefts remained when only isolate cases diagnosed without anomalies in other systems (n = 379) were included (Additional file 1: Tables S2–S4).

## Discussion

In this study, we investigated the associations between gaseous air pollutants exposure during the first trimester of pregnancy and the risk of orofacial clefts in Changsha, China using a case–control study design. We found positive effects of CO, NO_2_ and SO_2_ exposure on orofacial clefts independent of particulate matter (PM_2.5_ and PM_10_). While no significant association between O_3_ and orofacial clefts was found.

In the single pollutant analysis of CO, the risk of CL/P increased by 39–48% with a IQR increase in CO during the first trimester of pregnancy. The association was of comparable magnitude with a prospective cohort study including 133 cases of orofacial clefts from Wuhan, China, in which Zhao et al. observed that per 100 μg/m^3^ increase in CO increased the risk of CL/P in the second and the third month of pregnancy, with aORs of 1.31 (95% CI 1.14, 1.51) and 1.17 (95% CI 1.03, 1.33), respectively [15]. In contrast, another cohort study performed by Zhu et al. in the U.S. found significant effect of CO exposure on CPO (aOR = 2.74, 95% CI 1.62, 4.62), but not on CL/P during gestational weeks 3–8 [11]. Some other studies from different countries or regions also assessed the effect of CO on orofacial clefts, while observed insignificant [6, 24, 25] or reverse associations [26, 27].

In the single pollutant analysis of NO_2_, we observed increases of 35–61% in the risk of CL/P, as well as increases of 60–66% in the risk of CPO per IQR increase in NO_2_ in all months of the first trimester of pregnancy. Our estimates supported the results from the studies performed by Zhu et al. in the U.S. [11] and Wang et al. in China [18], which reported an aOR of 3.64 (95% CI 1.73, 7.66) for CPO per IQR increase during weeks 3–8 of pregnancy and a RR of 1.19 (95% CI 1.03, 1.36) for cleft palate per 10 μg/m^3^ increase during the first trimester of pregnancy, respectively. No other previous studies that estimated the effect of NO_2_ found significant results [18, 26, 28].

In the single pollutant analysis of SO_2_, we observed consistent and positive effects on CL/P during the period studied, with aORs ranging from 1.22 to 1.35. Similarly, in a case–control study with 145 cases from Australia, Hansen et al. found per IQR increase in SO_2_ exposure during weeks 3–8 of pregnancy was associated with elevated risk of CL/P (aOR = 1.27, 95% CI 1.01, 1.62) [24]. Besides, Marshall et al. in a study from New Jersey also provided evidence for the association between CL/P and SO_2_ at the highest exposure level (aOR = 1.6, 95% CI 1.1, 2.2) [26]. However, another study performed by Liu et al. in Liaoning, China reported no significant effects [17].

O_3_ is a secondary pollutant formed through reactions of volatile organic compounds and NO_2_ in the presence of sunlight [29], and its association with orofacial clefts has been investigated in a larger number of epidemiological studies compared to other gaseous air pollutants. However, only a few of them found positive results. For instance, a case–control study including 653 cases from Taiwan, China reported the risk of CL/P was increased in relation to O_3_ levels in the first (aOR = 1.20, 95% CI 1.02, 1.39) and the second month of pregnancy (aOR = 1.25, 95% CI 1.03, 1.52) [6], Another cohort study performed by Zhao et al.in Wuhan, China announced significant associations for CPO in the second and the third month of pregnancy, with aORs of 1.21 (95% CI 1.03, 1.42) and 1.18 (95% CI 1.02, 1.37), respectively [15]. Unfortunately, we failed to detect a positive effect of O_3_ on CL/P or CPO in Changsha, China in this study, as did most of the previous studies [10, 12, 18].

In our study area, the concentrations of air pollutants were higher than in areas covered by most of the previous studies, especially those from developed countries. As a result, it is reasonable to partially attribute our obvious findings to the differences in the level and the range of air pollutants across studies. Other factors, such as differences in the coverage of region and time period, statistical method used, covariates adjusted for, and characteristics of the subjects across studies may also result in mixed results [15]. Besides, we noticed that the effects of NO_2_ on CL/P were stronger than those of CO and SO_2_. One possible reason is that NO_2_ has stronger toxic effect on the development of CL/P, another is that the population studied were more sensitive to NO_2_ exposure. More animal studies comparing the toxic effects of different air pollutants are warranted to address this hypothesis. What is more, only NO_2_ exposure was observed to be associated with increased risk of CPO. Although the embryological pathogens of CL/P and CPO were different [1], the null associations between other air pollutants and the CPO subgroup may also result from the lack of sufficient statistical power with small case numbers.

The question of whether the observed associations for gaseous air pollutants were independent of particulate matter is important for health-risk assessment. Based on our analysis, the magnitudes of the effects of CO, NO_2_ and SO_2_ remained significant and even increased in the two-pollutant models, and the VIFs were all < 2, which provides evidence for independent effects of the three pollutants. Two previous studies have built co-pollutants models to adjust for coexistent pollutants for O_3_ and PM_10_, respectively [6, 30]. Although both of the studies observed increased effects of the air pollutants studied compared to single-pollutant models, which is in lines with our findings, neither provided plausible explanations for the interesting patterns. We hypothesize that both particulate matter and gaseous pollutants may be involved in the same pathways contributing to the development of orofacial clefts, and thus generate competitive relationships. As a result, the effects of gaseous pollutants were exacerbated after we controlled for PM in the models. However, more efforts are needed to test if the observed patterns are real.

The biological mechanism underlying the associations between gaseous air pollutants and orofacial clefts remains unclear. Data from animal studies provided several explanations for the potential teratogenicity of air pollutants. For instance, exposure to CO could lead to hypoxia [31, 32], reaction with hem-containing proteins [31, 33], and a reduction in metabolization of xenobiotics [27, 34], these responses can further trigger the fetotoxic effects even at a low concentration level [35]. Exposure to NO_2_ might cause the development of orofacial clefts by inducing the generation of inflammatory response or epigenetic changes, such as DNA methylation [36–38]. With respect to SO_2_, some studies suggest it could cause oxidative damage and induce multiple organ malformations in mice [39]. Although we did not observe significant effect for O_3_, there is evidence from an animal study indicating O_3_ exposure could act as a toxin to influence the fetal development in rats through hemodynamic, anoxic events, oxidative stress, and toxicity to certain cell populations [5]. More studies are needed to address the knowledge gaps.

To the best of our knowledge, the present study is one of the few studies to investigate associations between gaseous air pollutants exposure during the first trimester of pregnancy and risk of orofacial clefts in China. Our study would be in a better position to investigate this issue for three major reasons. We used the latest diagnosed cases and air pollution data from an area with high incidence of birth defect and higher levels of air pollution compared to developed countries. The results will be relevant for public health in areas with similar air pollution concentrations. We constructed two-pollutant models, and calculated VIFs to distinguish the independent effects of gaseous air pollutants, which made our results more persuasive. We included stillbirths, dead fetuses and live-born infants with orofacial clefts in the study area, which reduced potential selective bias compared to those studies which included live-born cases only.

Limitations in our study should be acknowledged. As in most previous researches, misclassification bias in the exposure estimation for individuals based on the nearest monitoring station approach was inevitable. However, the misclassification has been proven to be random and make the results bias towards the null [40]. We have no data on certain potential confounding factors, such as maternal occupational exposures, maternal nutrient, alcohol use and smoking during early pregnancy. Although the prevalence of smoking among Chinese women is quite low overall [41], and there is evidence from the U.S. showing that the risk of air pollutants on adverse outcomes changed by less than 5% after adjusting for occupation, income, maternal smoking and environmental tobacco smoke, and alcohol drinking [42], we cannot conclude that the absence of such information would not greatly change our results because the characteristics of populations in the current study may differ from those in developed countries (i.e. high environmental tobacco smoke prevalence among Chinese women[43]). Because of all the limitations mentioned above, the findings should be interpreted cautiously before extension to other populations and researches.

## Conclusion

Our study suggested that maternal exposure to CO, NO_2_, and SO_2_ during the first trimester of pregnancy were associated with increased risk of orofacial clefts, and the effects of the three gaseous air pollutants were potentially independent of PM_2.5_ and PM_10_. Our findings highlights the importance of addressing the effects of gaseous air pollutants on orofacial clefts in Changsha, China.

## Supplementary Information

**Additional file 1.** Supplementary materials.

## Data Availability

The datasets generated and/or analysed during the current study are not publicly available due ethical reason but are available from the corresponding author on reasonable request.

## Abbreviations

CL/P: Cleft lip with or without cleft palate
CPO: Cleft palate only
VIF: Variance inflation factor
PM: Particulate matter
IQR: Interquartile range
aOR: Adjusted odds ratio
CI: Confidence interval
