# Supplementary material for: First trimester exposure to ambient gaseous air pollutants and risk of orofacial clefts: a case–control study in Changsha, China
Source: BMC Oral Health. 2021 Oct 15;21:530. doi: 10.1186/s12903-021-01876-7 (PMC8518237; doi:10.1186/s12903-021-01876-7)
Supplement: Supplementary file 1 — Additional file 1. Supplementary materials. [file 12903_2021_1876_MOESM1_ESM.docx]

| Table S1.Correlations of air pollutants concentrations during 2015-2018 in Changsha, China. | | | | | | |
| --- | --- | --- | --- | --- | --- | --- |
|  | CO | NO_2_ | SO_2_ | O_3_ | PM_2.5_ | PM_10_ |
| CO | 1.00 | 0.67 | 0.41 | -0.31 | 0.73 | 0.63 |
| NO_2_ |  | 1.00 | 0.56 | -0.14 | 0.69 | 0.71 |
| SO_2_ |  |  | 1.00 | 0.16 | 0.50 | 0.63 |
| O3 |  |  |  | 1.00 | -0.08 | 0.10 |
| PM_2.5_ |  |  |  |  | 1.00 | 0.91 |
| PM_10_ |  |  |  |  |  | 1.00 |
| Notes: All *P* values of Spearman correlation coefficients <0.001. | | | | | | |

| Table S2. Adjusted odds ratios (95% confidence intervals) for orofacial clefts during the first trimester of pregnancy after excluding the cases diagnosed with anomalies in other systems. | | | |
| --- | --- | --- | --- |
| Air pollutant/  Time scale | Overall cases  (n=379) | Cleft lip with or without cleft palate  (n=317) | Cleft palate only  (n=62) |
|  | aOR(95%CI) | aOR(95%CI) | aOR(95%CI) |
| per IQR increase in CO |  |  |  |
| 1^st^ Month | 1.34(1.14,1.57) | 1.41(1.18,1.67) | 0.96(0.62,1.47) |
| 2^nd^ Month | 1.33(1.13,1.55) | 1.40(1.19,1.66) | 0.98(0.64,1.51) |
| 3^rd^ Month | 1.43(1.22,1.67) | 1.51(1.27,1.79) | 0.89(0.57,1.39) |
| per IQR increase in NO_2_ |  |  |  |
| 1^st^ Month | 1.54(1.31,1.81) | 1.58(1.33,1.88) | 1.26(0.83,1.91) |
| 2^nd^ Month | 1.50(1.28,1.77) | 1.51(1.27,1.81) | 1.30(1.01,1.96) |
| 3^rd^ Month | 1.38(1.18,1.63) | 1.36(1.14,1.62) | 1.36(1.01,2.02) |
| per IQR increase in SO_2_ |  |  |  |
| 1^st^ Month | 1.23(1.03,1.47) | 1.24(1.02,1.49) | 1.04(0.64,1.69) |
| 2^nd^ Month | 1.28(1.08,1.51) | 1.31(1.09,1.57) | 0.92(0.56,1.50) |
| 3^rd^ Month | 1.28(1.08,1.53) | 1.35(1.11,1.63) | 0.95(0.58,1.55) |
| per IQR increase in O_3_ |  |  |  |
| 1^st^ Month | 0.89(0.72,1.10) | 0.85(0.68,1.07) | 1.20(0.71,2.02) |
| 2^nd^ Month | 0.92(0.75,1.13) | 0.89(0.71,1.12) | 1.03(0.61,1.72) |
| 3^rd^ Month | 1.11(0.90,1.36) | 1.13(0.90,1.43) | 1.00(0.60,1.65) |
| Notes: aOR, adjusted odds ratio; CI, confidence interval. In the models of overall cases and CL/P, adjusted covariates including maternal age, maternal educational level, gravidity, infant sex, plurality, temperature and relative humidity. In the model of CPO, only maternal age, maternal educational level, gravidity, plurality, temperature and relative humidity were adjusted. | | | |

| Table S3. Adjusted odds ratios (95% confidence intervals) for orofacial clefts after additionally adjusting for PM_2.5_ during the first trimester of pregnancy after excluding the cases diagnosed with anomalies in other systems. | | | |
| --- | --- | --- | --- |
| Air pollutant/  Time scale | Overall cases  (n=379) | Cleft lip with or without cleft palate  (n=317) | Cleft palate only  (n=62) |
|  | aOR(95%CI) | aOR(95%CI) | aOR(95%CI) |
| per IQR increase in CO |  |  |  |
| 1^st^ Month | 1.43(1.15,1.76) | 1.51(1.20,1.89) | 0.85(0.48,1.50) |
| 2^nd^ Month | 1.52(1.23,1.87) | 1.63(1.30,2.04) | 1.11(0.61,2.00) |
| 3^rd^ Month | 1.80(1.46,2.22) | 1.92(1.53,2.41) | 0.95(0.51,1.75) |
| per IQR increase in NO_2_ |  |  |  |
| 1^st^ Month | 1.92(1.53,2.41) | 1.93(1.51,2.47) | 1.43(0.79,2.58) |
| 2^nd^ Month | 1.91(1.53,2.39) | 1.88(1.48,2.40) | 1.91(1.06,3.41) |
| 3^rd^ Month | 1.71(1.38,2.13) | 1.59(1.26,2.02) | 1.98(1.15,3.41) |
| per IQR increase in SO_2_ |  |  |  |
| 1^st^ Month | 1.19(0.98,1.46) | 1.18(0.96,1.46) | 1.00(0.57,1.73) |
| 2^nd^ Month | 1.28(1.07,1.53) | 1.30(1.08,1.58) | 0.96(0.56,1.61) |
| 3^rd^ Month | 1.30(1.08,1.56) | 1.36(1.11,1.66) | 0.99(0.59,1.66) |
| per IQR increase in O_3_ |  |  |  |
| 1^st^ Month | 0.98(0.76,1.26) | 0.94(0.71,1.24) | 1.41(0.75,2.64) |
| 2^nd^ Month | 0.95(0.74,1.24) | 0.93(0.70,1.24) | 0.93(0.49,1.77) |
| 3^rd^ Month | 1.22(0.94,1.58) | 1.32(0.99,1.75) | 0.87(0.46,1.64) |
| Notes: aOR, adjusted odds ratio; CI, confidence interval. In the models of overall cases and CL/P, adjusted covariates including maternal age, maternal educational level, gravidity, infant sex, plurality, temperature, relative humidity and PM_2.5_. In the model of CPO, only maternal age, maternal educational level, gravidity, plurality, temperature, relative humidity and PM_2.5_ were adjusted. | | | |

| Table S4. Adjusted odds ratios (95% confidence intervals) for orofacial clefts after additionally adjusting for PM_10_ during the first trimester of pregnancy after excluding the cases diagnosed with anomalies in other systems. | | | |
| --- | --- | --- | --- |
| Air pollutant/  Time scale | Overall cases  (n=379) | Cleft lip with or without cleft palate  (n=317) | Cleft palate only  (n=62) |
|  | aOR(95%CI) | aOR(95%CI) | aOR(95%CI) |
| per IQR increase in CO |  |  |  |
| 1^st^ Month | 1.39(1.17,1.66) | 1.44(1.19,1.74) | 1.01(0.62,1.63) |
| 2^nd^ Month | 1.35(1.13,1.60) | 1.43(1.19,1.72) | 1.08(0.66,1.76) |
| 3^rd^ Month | 1.49(125,1.77) | 1.54(1.28,1.86) | 0.94(0.58,1.52) |
| per IQR increase in NO_2_ |  |  |  |
| 1^st^ Month | 1.78(1.47,2.16) | 1.78(1.45,2.20) | 1.56(0.94,2.59) |
| 2^nd^ Month | 1.69(1.38,2.05) | 1.68(1.35,2.08) | 1.81(1.06,3.08) |
| 3^rd^ Month | 1.52(1.25,1.85) | 1.41(1.06,1.75) | 1.68(1.06,2.67) |
| per IQR increase in SO_2_ |  |  |  |
| 1^st^ Month | 1.27(1.03,1.55) | 1.23(0.99,1.53) | 1.15(0.66,1.98) |
| 2^nd^ Month | 1.29(1.07,1.56) | 1.32(1.08,1.61) | 1.01(0.58,1.75) |
| 3^rd^ Month | 1.33(1.09,1.62) | 1.35(1.09,1.69) | 1.05(0.60,1.81) |
| per IQR increase in O_3_ |  |  |  |
| 1^st^ Month | 0.90(0.73,1.12) | 0.87(0.69,1.10) | 1.17(0.68,2.01) |
| 2^nd^ Month | 0.94(0.76,1.17) | 0.92(0.73,1.16) | 0.98(0.58,1.67) |
| 3^rd^ Month | 1.14(0.92,1.41) | 1.19(0.94,1.51) | 0.96(0.57,1.61) |
| Notes: aOR, adjusted odds ratio; CI, confidence interval. In the models of overall cases and CL/P, adjusted covariates including maternal age, maternal educational level, gravidity, infant sex, plurality, temperature, relative humidity and PM_10_. In the model of CPO, only maternal age, maternal educational level, gravidity, plurality, temperature, relative humidity and PM_10_ were adjusted. | | | |
